# Alcohol as a Therapeutic Agent

**Published:** 1913-11

**Authors:** 


Alcohol as a Therapeutic Agent.—The exact pharmacological classification of alcohol, so long and so universally regarded as a typical stimulant, is no doubt at the present time considered by most to be a matter *sub judice*. It is a well known fact, however, that during the last few years there has been accumulating a mass of experimental testimony, from skilled investigators, which indicates that it is a narcotic, rather than a stimulant, and some extremists, holding that it is an anesthetic and depressing toxine, which in disease destroys the normal activities of the nerve cells and centers, would banish its use from medicine altogether. It is also a fact that in recent years its employment as a therapeutic agent has become very considerably restricted, as compared with former usage. But, on the other hand, it is unquestionably the case that among clincians generally alcohol is still held in the highest esteem in certain conditions, in some of which no other agent appears to be equally efficient. Thus, at one of the recent meetings of the Americcan Therapeutic Society, when Doctor

Crothers read a paper in which he characterized the use of alcohol in medicine as a "delusion founded on misconception and false reasoning, with the accumulated prejudices of ages," Doctor Alexander Blackader, professor of therapeutics in McGill University, in discussing it, said he would like to place himself on record as "belonging to the old school who still think they see benefit not infrequently arising from the use of alcohol as a prompt, although fleeting stimulant. In prolonged pyrexia it conserves nutrition and is utilized as a food. The time has not yet come when we can altogether dispense with alcohol in our pharmacopeias." Whatever the results of laboratory investigations may be, certainly the weight of clinical experience cannot be disregarded.

In *American Medicine*, for September, Doctor A. Jacobi states that no amount of whiskey will lead to intoxication when its effect is needed to combat sepsis and that his cases of thorough sepsis relieved or cured by alcohol extend over more than half a century. Among these were cases of diphtheria with mixed infection, where his experience had shown that no such infection was amenable to the action of antitoxine. The late Austin Flint, than whom we have never had a physician of more extended clinical experience or keener observation, was throughout his long career a strong advocate of the judicious use of alcohol. He never advised it unless there were present what he believed to be distinct indications for its employment, but in cases where such were present he did not hesitate to prescribe it in enormous quantities. In his *Practice* he records instances in which desperate, and even apparently hopeless, cases were undoubtedly saved, like some of those cited by Doctor Jacobi, by this course.

There would seem to be little doubt, in the light of the later investigations, that alcohol may act as a narcotic; but the acceptance of this theory is by no means equivalent to condemning its therapeutic use for its effects on the brain. On the contrary, Cushny maintains that this depressant action, far from being in conflict with its clinical employment, supplies a definite and logical explanation of the improvement noted in a large number of instances where the effect of alcohol in allaying the subjective symptoms, relieving the nervous strain, and promoting the rest and comfort of the patient is not surpassed by that of any other drug. Accordingly, he says, it would seem a question whether the results aimed at by the clinician when he prescribes alcohol have not been misnamed "stimulation" (the word being used in quite a different sense from that in which it is understood by the experimental observer), and are not in reality narcotic in their nature, and hence in entire agreement with the experimental results.—*Ed. in N. Y. Medical Journal.*

---

METAMORPHOSES OF PRIMARY SKIN EFFLORESCENES.—K. Herxheimer noticed in a great number of cases how primary blebs and pustules in syphilis, Dühring's disease, herpes progenitalis, labialis, and perianalis, varicella, eczema, urticaria recidva (strophulus infantum), and simple pyodermia underwent change into lichenoid papules; evidently an expression o nthe part of the organism to transmute an acute inflammation, which would not subside rapidly into a more chronic process.—*Ex.*

---

THE VALUE OF THE CYANIDE OF GOLD AND POTASSIUM IN THE TREATMENT OF LUPUS VULGARIS AND ERYTHEMATODES.—The results obtained from the use of the cyanide of gold and potassium combined with tuberculin in the treatment of lupus are not entirely satisfactory, but admit of improvement. On the other hand good scar tissue was obtained with gold, tuberculine, and pyrogallus.—*Ex.*

---

INCREASED MORTALITY OF INFANTS IN SPRING.—H. Liefmann observes that the increased mortality during the warm days of spring is not due so much to the spoiled milk as to the direct injury of the infants by the heat, boys being more susceptible to it than girls.—*Ex.*

